# Tumor Microenvironment and Glioblastoma Cell Interplay as Promoters of Therapeutic Resistance

**DOI:** 10.3390/biology12050736

**Published:** 2023-05-18

**Authors:** Edoardo Agosti, Pier Paolo Panciani, Marco Zeppieri, Lucio De Maria, Francesco Pasqualetti, Alessandro Tel, Luca Zanin, Marco Maria Fontanella, Tamara Ius

**Affiliations:** 1 Division of Neurosurgery, Department of Medical and Surgical Specialties, Radiological Sciences and Public Health, University of Brescia, Piazzale Spedali Civili 1, 25123 Brescia, Italy; 2 Department of Ophthalmology, University Hospital of Udine, Piazzale S. Maria della Misericordia 15, 33100 Udine, Italy; 3 Division of Radiation Oncology, Azienda Ospedaliero Universitaria Pisana, 56100 Pisa, Italy; 4 Department of Oncology, University of Oxford, Old Road Campus Research Building, Roosevelt Drive, Oxford OX3 7DQ, UK; 5 Clinic of Maxillofacial Surgery, Head-Neck and NeuroScience Department, University Hospital of Udine, Piazzale S. Maria della Misericordia 15, 33100 Udine, Italy; 6 Neurosurgery Unit, Head-Neck and NeuroScience Department, University Hospital of Udine, Piazzale S. Maria della Misericordia 15, 33100 Udine, Italy

**Keywords:** tumor microenvironment, stem cells, tumor-associated macrophages, chemoresistance, radioresistance, temozolomide, M2-macrophages, non-coding RNA, immunotherapy

## Abstract

**Simple Summary:**

Despite years of molecular discoveries and technological advances in surgery, the prognosis of glioblastoma (GBM) still remains unfavorable, with a mean overall survival typically less than 20 months. Recurrence of tumors, and specifically of GBM, could be due to the persistence of a subpopulation of cancer cells with stem cell characteristics. Current investigations have shown the importance of the tumor microenvironment (TME) and its interplay with GBM stem cells through the release of extracellular vesicles. Mechanisms (e.g., M2-macrophages polarization, immunosuppression), factors (cytokines and chemokines), and key players in the TME have a potential role in GBM recurrence, and could be an ideal target for new therapeutic approaches to this highly aggressive tumor. The aim of this short review is to assess the current literature regarding TME, which specifically deals with the interaction between GBM cells and resident tumor-associated macrophages, microglia, lymphocytes, and the implicated role of extracellular vesicles. A better understanding of the interactions between GBM cells, other cells, and factors in the TME can help in investigating the mechanisms underlying chemo- and radioresistance, and in the discovery of new therapeutic approaches to treat and prevent disease progression in GBM patients.

**Abstract:**

The invasive nature of glioblastoma is problematic in a radical surgery approach and can be responsible for tumor recurrence. In order to create new therapeutic strategies, it is imperative to have a better understanding of the mechanisms behind tumor growth and invasion. The continuous cross-talk between glioma stem cells (GSCs) and the tumor microenvironment (TME) contributes to disease progression, which renders research in this field difficult and challenging. The main aim of the review was to assess the different possible mechanisms that could explain resistance to treatment promoted by TME and GSCs in glioblastoma, including the role of M2 macrophages, micro RNAs (miRNAs), and long non-coding RNAs (lncRNAs) from exosomes from the TME. A systematic review of the literature on the role of the TME in developing and promoting radioresistance and chemoresistance of GBM was performed according to PRISMA-P (Preferred Reporting Items for Systematic Review and Meta-Analysis Protocols) guidelines. A dedicated literature review search was also performed on the immunotherapeutic agents against the immune TME. We identified 367 papers using the reported keywords. The final qualitative analysis was conducted on 25 studies. A growing amount of evidence in the current literature supports the role of M2 macrophages and non-coding RNAs in promoting the mechanisms of chemo and radioresistance. A better insight into how GBM cells interact with TME is an essential step towards comprehending the mechanisms that give rise to resistance to standard treatment, which can help to pave the way for the development of novel therapeutic strategies for GBM patients.

## 1. Introduction

Glioblastoma (GBM) is the most prevalent brain tumor in adults, and despite multimodality therapy, including maximal safe resection surgery, chemotherapy (CTX), and radiotherapy (RT), the prognosis tends to be rather unfavorable with an average overall survival (OS) typically less than 20 months [1,2,3,4]. The tumor’s site and genetic and molecular profiles, such as IDH1 mutation, p53 mutation, 1p/19q codeletion, EGFR amplification, MGMT methylation, and ATRX mutation, play a crucial role in defining its prognosis. [3,4,5,6].

A phase III trial study by Stupp et al. in 2005 [1] reported the important role of TMZ with concurrent RT followed by adjuvant treatment with TMZ in the treatment of newly diagnosed GBM, and no other CT drug has demonstrated notably better results. Additionally, although bevacizumab has U.S. Food Drug Administration (FDA) approval for recurrent GBM (rGBM), no phase III clinical trials have proved its OS benefits. Consequently, no cure for GBM exists. Elevated dismal outcomes are primarily due to the tumor’s high recurrence rate, which is closely tied to its resistance to standard therapies. Studies have proposed different therapeutic alternatives in the management of patients with GBM, however, a gold standard approach is still lacking [3,6,7].

Recurrence of the tumor in GBM individuals is one of the main causes of mortality, which could be due to the presence and persistence of cancer cells with stem cell characteristics [3,6,7,8,9]. Over the past ten years, researchers have successfully isolated glioblastoma stem cells (GSCs) from GBM and discovered their critical role in the development, maintenance, and recurrence of tumors. As a result, GSCs have become a crucial target for new treatments due to their ability to resist standard therapies and contribute to malignant relapse [3,6,10,11].

An increased number of investigations have demonstrated that subpopulations of tumor stem cells can favor resistance to therapies and cause malignant relapse, due to their capabilities of differentiation, self-renewal, growth, and progression [3,6,8,9]. The resistance mechanisms of GSCs include their quiescence, higher mitochondrial reserve, extensive DNA repair capabilities, and location in hypoxic niches [11,12,13,14,15]. Simultaneously, numerous studies have shown that tumor microenvironment (TME) and its interplay with GBM stem cells, through the release of extracellular vesicles, is gaining an important role in GBM pathogenesis and proliferation characteristics. TME has been proposed as a promising therapeutic target [9,12,16,17,18].

The GBM microenvironment is a dynamic entity comprised of several different types of cells and factors, which include tumor cells, GSCs, endothelial cells, astrocytes, microglia, stromal components, soluble factors, extracellular matrix, and other substances [9,18]. Each component plays an important and specific role in the microenvironment. It has been shown that GSCs, for example, can establish a continuous cross-talk that actively assists in the remodeling of the microenvironment. These factors can be influential in maintaining and contributing to the progression of the disease [6,8,9,11,12]. Identifying the key actors in this scenario has become increasingly challenging, as the dialogue between GSCs and the microenvironment plays a significant role in supporting GBM [9,10,11,12,13,14,15,16]. The TME also includes immune and myeloid cells. Myeloid cells are the most frequent and, among them, tumor-associated macrophages (TAMs) are the most abundant [3,6,14,17,19,20].

Characterization of chemoresistance and radioresistance has traditionally been focused on the effects of TMZ and RT on the tumor cells, while overlooking the impact on the TME. Although components of the TME have been shown to regulate cell proliferation and migration, stemness maintenance, angiogenesis, and malignant progression, their role concerning the response to TMZ and RT in GBM has been less analyzed [5,9,15,16,20,21,22,23,24].

In this short review, we describe and discuss the reported mechanisms of chemo-radioresistance promoted by TME and GSCs in GBM, highlighting the role of M2 macrophages, micro RNAs (miRNAs), and long non-coding RNAs (lncRNAs) derived from exosomes from the TME, and exploring new possibilities of personalized CTX and RT treatments to improve patient outcomes.

## 2. Materials and Methods

This study was conducted in accordance with the PRISMA-P (Preferred Reporting Items for Systematic Review and Meta-Analysis Protocols) guidelines [25]. A systematic review of the literature on the role of the TME in developing and promoting radioresistance and chemoresistance of GBM was performed. An online literature search was launched on PubMed, Medline, Web of Science, and Scopus using the following research string: “((Glioma associated macrophages OR GAM OR Microglia) OR (glioblastoma tumor microenvironment OR TME) AND (radioresistance OR temozolomide resistance))”. Additionally, a dedicated literature review search was performed on the immunotherapeutic agents against the immune TME by launching on PubMed, Medline, and Scopus using the following research string: “((Glioma associated macrophages OR GAM OR Microglia) AND (immunotherapy OR immunotherapeutic agents))”.

**Figure 1:**
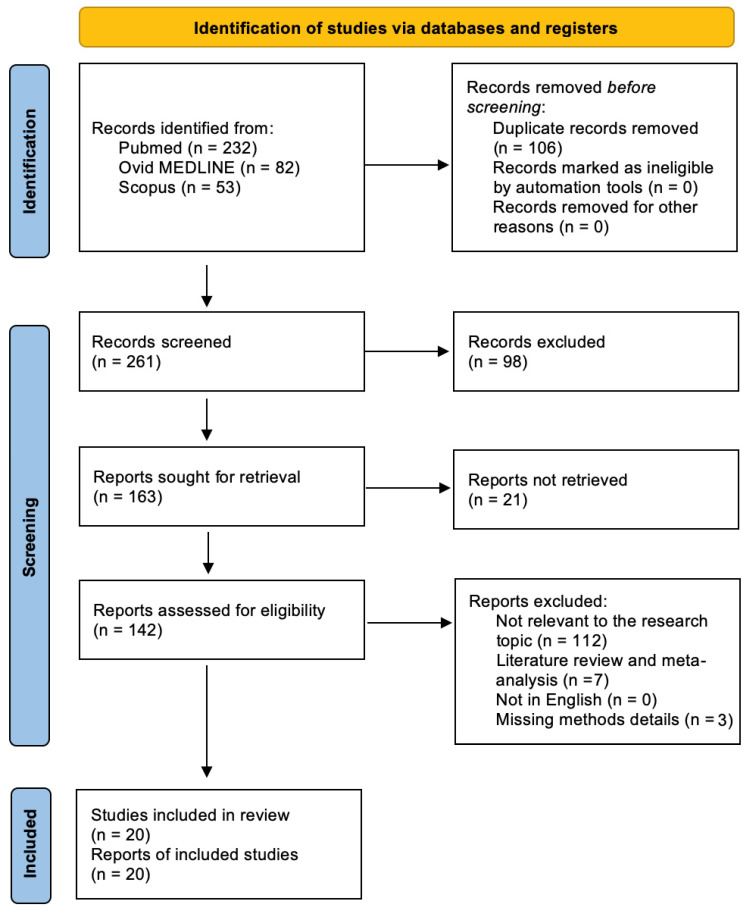
PRISMA diagram showing the research strategy and selection of papers about TME and its role in GBM radioresistance and chemoresistance.

In this study, exclusion criteria were established to identify eligible articles related to the topic of TME and its role in treatment resistance. These criteria included missing crucial methodological details and literature reviews, as well as the exclusion of case reports, editor letters, non-systematic reviews, and studies published in languages other than English. A literature search was conducted on 13 December 2022, and 367 papers were initially identified using relevant keywords. After removing duplicates and conducting abstract screenings, 142 full-text articles were obtained, and the final analysis was conducted on 20 studies that met the inclusion criteria, as shown in Figure 1 following PRISMA guidelines.

The search for pertinent articles was conducted up till 14 December 2022. The coauthors (L.D.M. and E.A.) independently performed the screening of abstracts for eligibility. Discordance between authors was resolved by the consensus of senior authors (P.P.P. and T.I.). There were no publication date restrictions. Articles regarding extracerebral neoplasms, metastases, or other types of brain cancer were not included. Exclusion criteria were as follows: letters to the editor, case reports, non-systematic reviews, retrospective cohort studies, manuscripts not published in English, papers missing paramount methods details, meta-analysis, and literature review.

## 3. Results

### 3.1. Literature Search

#### TME and Its Role in Radioresistance and Chemoresistance

A total of 367 papers were selected based on the keywords. Upon excluding 106 duplicates, the remaining 261 abstracts were assessed, which gave rise to 142 full-text articles that were eligible. A total of 122 studies that were not in accordance with the inclusion criteria were excluded. The final qualitative analysis was conducted on 20 studies. The selection study process is summarized in Figure 1, following PRISMA guidelines.

### 3.2. Included Studies

The reviewed papers were further divided into two categories based on the type of adjuvant therapy for which resistance was developed: (1) CTX and (2) RT. Noteworthy reports in the literature on chemoresistance mainly focused on resistance to TMZ.

Most of the studies focused on two mechanisms of chemo-radioresistance:

1. Exosomal non-coding RNAs, including both micro RNAs (miRNAs) and long non-coding RNAs (lncRNAs);
2. M2 macrophage polarization.

Table 1 describes the number of studies in our analysis that describe the mechanisms with which the TME provides TMZ resistance to the GBM cells, while the mechanisms for RT resistance are summarized in Table 2.

As for the TMZ resistance, all studies (15) were conducted on cellular culture with the aid of immunochemistry (10; 66%), RT-qPCR (four; 27%), and genomics (four; 27%). The predominant cell lines studied were the human GBM (14; 83%), followed by the human and murine monocyte-macrophages or microglia (six; 40%). The main mechanism of chemoresistance was the development of exosomal non-coding RNAs (six; 40%), particularly miRNAs (three; 20%) and lncRNAs (three; 20%), and the occurrence of M2 macrophages polarization (five; 33%).

Regarding RT resistance, all studies (seven) were conducted on cellular culture, with the aid of immunochemistry in the majority of cases (five; 71%). The GBM cell lines were the most studied (six; 85%). The mechanisms of radioresistance were diverse, from loss of mutation to growth factors and interleukin production, M2 macrophage polarization, and others.

### 3.3. TME and Immunotherapeutic Strategies

**Figure 2:**
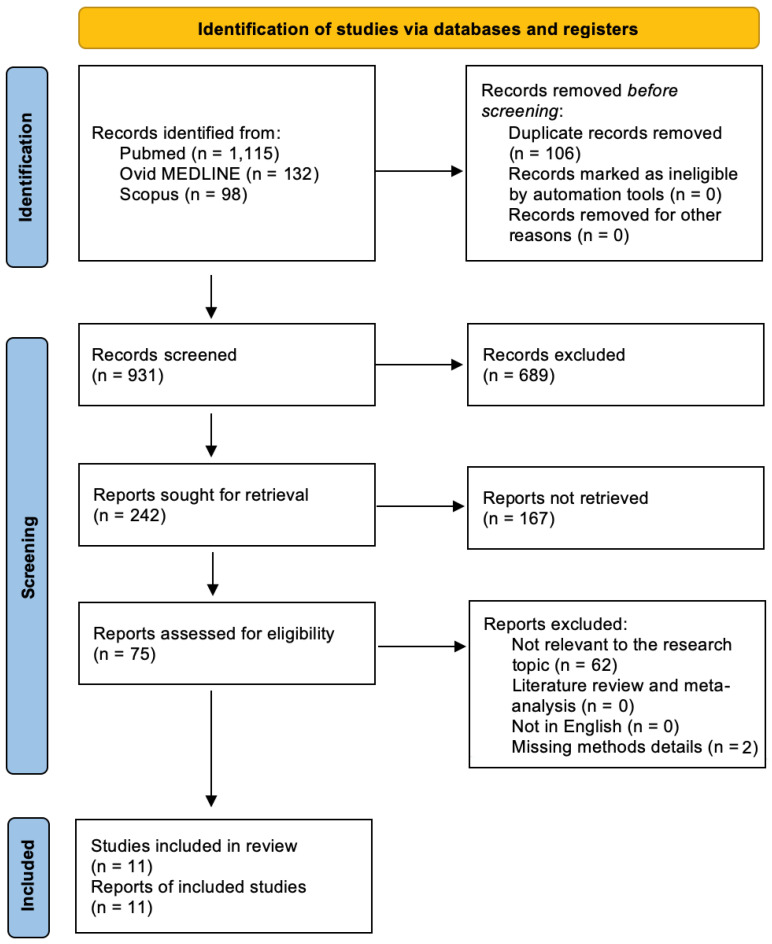
PRISMA diagram showing the research strategy and selection of papers about immunotherapeutic strategies for GBM.

Out of 1345 papers identified using the given keywords, 106 duplicates were removed and 931 abstracts were examined, resulting in 75 eligible full-text articles. After excluding 64 studies that did not meet the inclusion criteria, the final qualitative analysis was conducted on 11 studies. The paper selection based on PRISMA is shown in Figure 2.

Table 3 summarizes the studies describing the role of immunotherapeutic agents directed towards the GBM immune TME and the immunotherapeutic perspectives for GBM treatment that were actually published.

## 4. Discussion

**Figure 3:**
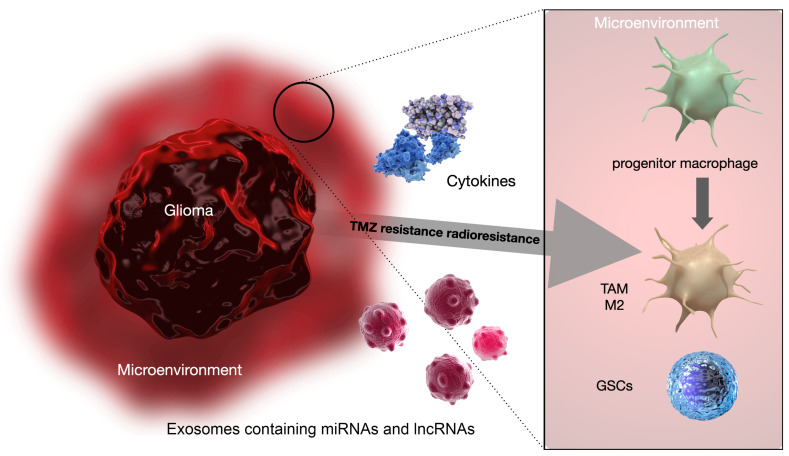
Schematic representation of the mutual communication between GSCs and GBM-TME through EVs. GSCs can release EVs directed toward the TME that carry various signaling molecules, including growth factors, cytokines, and chemokines, and promote tumor growth, angiogenesis, and immune suppression. GSCs can also communicate with each other through EVs containing miRNAs, lncRNAs, and other molecules. Similarly, cells in the TME can communicate with each other through EVs carrying cytokines, growth factors, and other signaling molecules. The cells in the TME, including immune cells, astrocytes, and endothelial cells, can also release EVs containing signaling molecules that can promote GSC self-renewal, differentiation, or chemo- and radioresistance. Understanding these pathways and the molecules involved in EV-mediated communication could provide new targets for the development of novel therapies for GBMs.

This systematic literature review aimed at investigating the mechanisms promoted by the TME for the acquisition of chemo-radioresistance by GBM cells. Several studies have reported that specific miRNAs’ and lncRNAs’ profiles and M2 macrophages’ polarization of the TME may have a considerable diagnostic and prognostic impact in promoting the GBM cells’ resistance to adjuvant therapies (Figure 3) [3,5,7,8,9].

TAMs are the major biological constituents of the GBM TME and are implicated in GBM progression, angiogenesis, and, according to recent reports, the development of resistance to adjuvant treatments [3,5,6]. GBM cells and TAMs are constantly in close cross-talk, communicating through paracrine signals, including cytokines and the extracellular production and release of exosomes, which are a novel class of extracellular vesicles that have gained enormous attention lately as facilitators of the progression of various tumors [18]. Exosomes are secreted by different cells in the body, including tumor cells and other cells in the tumor microenvironment, and are mainly used as vehicles to exchange information between cells. Their content in terms of nucleic acids (mainly DNA, mRNA, and miRNA) is dynamic and related to the cells of origin. Since each miRNA is responsible for the regulation of several mRNAs, the transport of different miRNAs from one cell to another via exosomes involves the regulation of several mRNAs in the acceptor cell and, as the final outcome, modifies its behavior. The exchange of information that follows the release of exosomes is capable of mutually modifying the phenotype of tumor cells and those in the tumor microenvironment. miRNAs and lncRNA can be transmitted via exosomes from cancer cells to other cells in the TME and vice versa, influencing tumor development, aggressiveness, and progression and TAM immunosuppressive polarization [18,21,22,23,24,26,47].

It has been currently postulated that TMZ resistance in GBM can be reflected by extracellular vesicles. Yin et al. [15] found that high serum levels of exosomes from GBM patients containing miRNA-1238 are indicative of TMZ resistance of the GBM cells. Chuang et al. [30] provided evidence that miRNA-21 and miR-416a enriched TAM M2-derived exosomes, contributing to GBM malignancy via increasing stemness and favoring the development of TMZ resistance. Witusik-Perkowska et al. [29] showed that miRNA-31, miRNA-221, miRNA-222, and miRNA-21 are expressed at higher levels in the serum of GBM patients with TMZ resistance. These miRNAs, downregulating PTEN expression in tumor cells, may reduce the sensitivity of GBM cells to TMZ. All this evidence leads to the assumption that specific miRNAs’ exosomal profiles (miRNA-31, miRNA-21, miRNA-221, miRNA-416a, miRNA-222, and miRNA-1238) in GBM patients’ serum could be considered as a possible biomarker in developing CTX protocols. Additional clinical data and studies, however, are needed to develop efficient, reliable, cost-effective, and convenient methods for identifying circulating miRNAs [15,18,21,22,23,24,26,29,30,47].

The role of miRNAs in favoring chemoresistance has been documented in current literature, however, there is less evidence regarding the role of lncRNAs. Wu et al. [28] demonstrated that lnc-RNA TALC could regulate M2 polarization and promote TMZ resistance in GBM by activating the p38 MAPK signaling pathway and promoting C5a release. In addition, Zhang et al. [23] demonstrated that exosome-mediated transfer of lncRNA 226 SBF2-AS1 from TAMs spreads TMZ resistance in GBM cells through a mechanism of upregulation of serum SBF2-AS1 levels, mediated by the transcription factor ZEB1, which binds directly to the SBF2-AS1 promoter region. Dai et al. [13] and Zheng et al. [36] showed that AHIF and lncRNAs linc-RA1 may contribute to GBM radioresistance. In detail, AHIF mediated radioresistance through VEGF-A and angiogenin in secreted exosomes [13]. On the other hand, lincRA1 stabilized the level of H2B K120 monoubiquitination (H2Bub1) by combining with H2B and inhibiting the interaction between H2Bub1 and ubiquitin-specific protease 44 (USP44), which inhibited autophagy, thus contributing to GBM radioresistance [36].

By the same mechanism, lnc-TALC also appears to favor the development of GBM radioresistance. The lncRNAs do not seem to play a role only in the development of TMZ resistance; they may also contribute to the development of GBM radioresistance. Zhang et al. [23] demonstrated that exosome-mediated transfer of lncRNA SBF2-AS1 from TAMs spreads TMZ resistance in GBM cells, while Dai et al. [13] and Zheng et al. [36] showed that lncRNAs linc-RA1 and AHIF may contribute to GBM radioresistance. This evidence suggests that the blocking of lncRNA-mediated cross-talk between GBM cells and TMAs might be a novel therapeutic strategy to address TMZ resistance and radioresistance. Moreover, specific lncRNA identifiers of an aggressive TME, predisposing to the development of TMZ resistance, can be searched for in the blood, as can miRNAs, as a biomarker of diagnosis of GBM and prognostic factor of adjuvant resistance therapies [13,23,28,36].

The specific identification and importance of exosomes in the microenvironment is still not completely known. Exploring the roles of intercellular communication via miRNAs and lncRNAs carried in exosomes is important for improving our understanding of the GBM biology and mechanisms involved in the development of resistance to adjuvant therapies [9,14,29,30,37,38,39,40,41,42,43,44,45,46,47,48,49].

TAMs can be categorized into two subtypes based on their function: M1 and M2 polarized macrophages. Studies have linked M2 macrophages to increased GBM aggressiveness by secreting various molecules, such as PDGF, EGF, TGF- β1, and VEGF, to surrounding cells. However, as shown more recently, M2 macrophages may also promote the TMZ resistance and radioresistance of GBM cells [8,11,16,47,48,49]. Recent data suggest that M2 macrophages may play an important role in regulating TMZ resistance. Zhang et al. [5] and Azambuja et al. [27] showed that VEGF-dependent M2 macrophages can activate the PI3K/Akt/Nrf2 pathway to favor TMZ resistance in individuals with GBM. These results agree with previous reports that showed that upregulated VEGF levels can be important in TMZ resistance. Chuang et al. [30] showed that eliminating M2 macrophage-derived miRNA-21 exosomes can overcome GB’s TMZ resistance. M2 macrophages have been shown to have the typical surface biomarker CD163. Miyazaki et al. reported that CD163-positive M2 macrophages could also play a role in the increased immune therapy resistance in GBM cells that show TMZ resistance [50].

Accordingly, M2-polarized macrophages promote cancer stemness and chemoresistance in other types of tumors, including pancreatic cancer and thyroid cancer. Thus, in light of the numerous data reported in the literature, it is reasonable to speculate that M2 macrophages may contribute to cancer stemness, progression, aggressiveness, and TMZ resistance in GBM [31,33].

M2 macrophages appear to have a pivotal role also in the development of GBM radioresistance. Jang et al. [37] reported that the M1/M2 macrophage ratio in the TME and radiosensitivity of GBM cells are inversely associated. In this regard, short-term relapse GBMs had a significantly higher fraction of M2 macrophages after RT compared with the long-term relapse tumors detected, suggesting that M2 macrophages may play a role in radioresistance and then develop an early relapse. Furthermore, several studies have suggested that immunotherapy targeting M2 macrophages may favor the radiosensitivity of GBM cells [27,34]. These first findings highlight the primary role of M2 macrophages in the development of GBM radioresistance. The increase of the M1/M2 ratio by conversion of M2 macrophages into M1 macrophages or the destruction of M2 macrophages may, thus, represent a potential therapeutic approach for increasing GBM radiosensitivity [27,35,47,48,49,50,51].

To summarize, a high proportion of M2 macrophages’ and specific miRNAs’ (including miR-1238, miRNA-31, miRNA-221, miRNA-222, miRNA-416a, and miRNA-21) and lncRNAs (lnc-TALC, SBF2-AS1, linc-RA1, and AHIF) profiles in the TME may play a pivotal role in the acquisition of TMZ resistance and radioresistance of GBM cells. Exosomal miRNA and lncRNA levels in human serum may function not only as a potential diagnostic biomarker in GBM patients, but also as a prognostic factor for the early identification of TMZ resistant and radioresistant GBMs [27,35,48,50].

**Figure 4:**
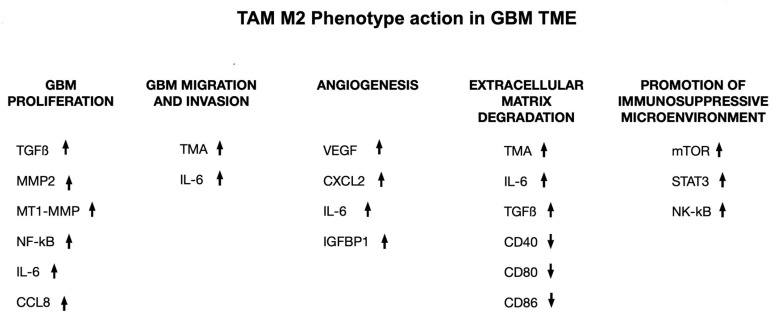
The transition of TAMs from M1 to M2 phenotype is linked to the advancement of tumors. TAMs are thought to drive the progression of GBM through various cytokines and factors, promoting proliferation of GBM cells, migration and invasion of GBM cells, angiogenesis within GBM, breakdown of the extracellular matrix (ECM), and an immunosuppressive TME. ↑ = increase in production; ↓ = reduction of production.

Macrophage-targeting immunotherapy has been proposed as a potential tool to improve GBM treatment strategies by preventing TAMs recruitment, repolarizing TAMs, and using immune checkpoint blockade [52]. M2-like phenotype TAMs tend to be important in the immunosuppressive TME, which can secrete immunosuppressive substances like TGF-b, IL-6, and IL-10 in GBM. The secreted pro-inflammatory cytokines include IL-12, IL-2, IFN-g, and TNF-a, which can be detected in low levels [53,54]. The M1 to M2 phenotype transition in TAMs tends to be associated with the progression of the tumor. TAMs have been shown to be important in the progression of GBM by pro-tumorigenic activities, which include GBM cell migration, proliferation, and invasion. TAMs have also been shown to play an important role in the activation of angiogenesis, generating immunosuppressive TME, and facilitating the degradation of the extracellular matrix (Figure 4) [55,56].

Several TAM substances, like stress-inducible protein (STI)-1, TGF-β, IL-1β, IL-6, and EGF, have been shown to enhance GBM cell invasion. Recent studies have shown that M2 macrophages play a role in promoting the vascularization of brain tumors by secreting pro-angiogenic factors like VEGF. As a result, TAM pathways have emerged as promising targets for immunotherapy. In vitro and in vivo analyses have shown that targeting these pathways can lead to reduced myeloid infiltrates, decreased tumor vascularity, and improved overall survival (Table 3) [56,57].

Accordingly, over the years, macrophage-targeting immunotherapy has been advocated for as a potential tool to improve GBM treatment strategies. In detail, three of the main macrophage-targeting immunotherapy strategies include: TAMs recruitment prevention, TAMs repolarization, and immune checkpoint blockade.

An effective strategy for treating GBM may involve preventing the recruitment of M2 macrophages to the GBM site, considering their role in tumor invasion and progression. Ongoing clinical trials are targeting this approach with inhibitors for the following targets: angiopoietin-2 (ANG2), CXCR4, and colony-stimulating factor 1 receptor (CSF1R), [58,59,60,61,62]. Another potential strategy is promoting a shift between M2 and M1 macrophages. CD40 and TLR agonists are currently being tested in GBM clinical trials as potential therapeutic agents targeting TAM repolarization [63,64,65,66]. In addition, immune checkpoint inhibitors such as anti-PD-1 and anti-CTLA-4 have shown success in treating melanoma and non-small cell lung cancer in recent years. Therefore, immune checkpoint blockade is a promising treatment that is also implicated in clinical trials for GBM (Table 4) [67,68].

### Future Perspectives

Because of the interaction between all parts of the brain network, the growth of GBM results in the invasion of healthy brain tissue. TME, a complex peritumoral environment created as a result, is made up of tumor cells as well as different non-tumor cells including nerve, stem, fibroblast, vascular, and immune cells. Since it affects the biological state of the tumor and increases its capacity to resist treatment, the microenvironment is a crucial contributor to the unsuccessful treatment of GBM. The interaction of GBM cells with TME promotes the growth and invasion of the tumor, as well as its resistance to therapy by impeding the effectiveness of molecular pathways. An enhanced knowledge of TME cell-to-cell interactions with tumoral cells can provide insights into more effective treatment options [69].

Targeting these factors could be a potential therapeutic approach for treatment, as shown by preliminary clinical trials, given the critical role of the cytokines produced by the TME and GBM cells in determining GBM malignancy, angiogenesis, proliferation, immunosuppression, chemo- and radioresistance. This leads to a more individualized approach to GBM therapy, frequently based on an immunotherapy strategy.

For example, GBM malignancy is linked to higher levels of IL-10 expression. Since STAT3 signaling is primarily responsible for the transcription of IL-10 in GAMs, overactive pSTAT3 expression has been linked to both a worse survival rate for GBM patients and an increase in tumor grade. According to some data, STAT3 targeting is a viable therapeutic strategy [39]. STAT3 suppression through short interfering RNA (siRNA) has been hypothesized to limit tumor growth. Since lncRNAs play an oncogenic function, immunotherapeutic drugs can also target them.

It has been demonstrated that elevated lncSNHG15 serum levels are linked to overexpression of oncogenesis-related genes such CDK6, EGFR, and Sox2. Additionally, higher levels of lncSNHG15 have been linked to substantial tumor aggressiveness in clinical GBM samples that were resistant to TMZ. The lncSNHG15/CDK6/miR-627 regulatory circuit has been implicated in the formation of GBM and polarization of GAMs in both in vitro and in vivo models, according to a preclinical study published in 2019 [70]. It has been demonstrated that CDK6 is overexpressed in GBM and is more prevalent in cells resistant to temozolomide. Palbociclib, a CDK6 inhibitor, was used to treat GBM tumorigenesis and the capacity to produce M2 GAM and GBM stem cells. The anti-GBM actions of Palbociclib have been linked to decreased levels of lncSNHG15 and increased levels of the tumor suppressor miR-627. Both LncSNHG15 knockdown and Palbociclib therapy resulted in a rise in TMZ sensitivity [43].

Reduced tumor angiogenesis can also be achieved through immunotherapy. By secreting pro-angiogenic factors like VEGF, for instance, GAMs aid in the vascularization of brain tumors. In a GBM mouse model, VEGFR blockage with the administration of Sunitinib (Sutent) and the VEGF inhibitor Bevacizumab (Avastin) prolonged survival, reduced myeloid infiltrates, and decreased tumor vascularity [39]. Additionally, GSCs possess a crucial genetic signature in the form of EGFR amplification and mutation. Monoclonal antibodies (mAbs) straightforwardly focusing on EGFR, for example, cetuximab, panitumumab, and nimotuzumab, are ordinarily utilized as a helpful methodology in GBM. By interfering with ligand binding and EGFR extracellular dimerization, these mAbs prevent EGFR-mediated signaling and may also cause EGFR receptor internalization and destruction [71].

In addition, cytokines in GBM hinder the production of anti-tumor immune responses and create an immunosuppressive microenvironment. As a result, therapies may attempt to reverse or lessen this state of inhibition. Programmed cell death-1 (PD-1) and cytotoxic T lymphocyte-associated protein antigen-4 (CTLA-4) are two of the most significant immune checkpoints. FDA-endorsed inhibitors of CTLA-4 and PD-1 (nivolumab) have been created, and their blend with radiotherapy and chemotherapy has been displayed to further develop endurance in some GBM patients (38). Additionally, immune cells like macrophages and microglia localize and migrate to tumors via chemokines. Tumor invasion is closely linked to the chemokines ligand 1 (CXCL1) and ligand 2 (CXCL2). Focusing on CXCL1/2 with standard chemotherapy can further develop the chemotherapy productivity of GMB and drag out the endurance of GBM in mice [43].

It has been hypothesized that some of the most important molecules can be targeted to enhance the response to adjuvant therapy, despite the fact that many molecules are involved in the development of chemo- and radioresistance. For instance, in a stage I preliminary on mouse models NSCCRAd-S-pk7 (a mix of oncolytic adenoviruses (CRAd-S-pk7) that target GSCs with Brain Undifferentiated organism) infusion has been demonstrated to be protected and compelling in patients with recently analyzed GBM during medical procedures. Chemoradiotherapy treatment efficiency can be improved through multiple-site injections into the brain. Another study [72] demonstrated that Zika virus can induce apoptosis, inhibit GSC proliferation, and promote chemo- and radiosensitivity by targeting GSC through the SOX2 integrin axis.

Moreover, GAMs are associated with the radio-obstruction of glioblastoma by discharging TNF α, which increments the atomic element κB (NF-κB) that is connected with substandard endurance. Different techniques have been considered to lessen the enlistment of GAMs in GBM movement, including adjusting the GAMs aggregate towards an enemy of growth M1-like microenvironment, or diminishing the M2-like cancer-advancing microenvironment (11). For GBM treatment, TLRs (toll-like receptors) could be new targets. They suppress pro-tumorigenic pathways by modulating immune responses in GBM and the innate immune system. Hence, they could address a possible new objective in GBM treatment. Polyinosinic-polycytidylic acid and poly-L-lysine (Poly ICLC—Hiltonol), a TLR3 agonist, can activate immune cells and encourage their migration into the tumor mass. In a stage I preliminary, recently analyzed GBM patients were given Poly ICLC in blend with standard consideration and customized peptides, in light of individual cancer transformations, as a growth explicit antigen immunization. Other immunostimulants, such as the TLR3 agonist Poly I, were also tested with Poly ICLC: C and TLR7/8 agonist Imiquimod in malignant growth antibody treatments on patients who went through complete growth resection of GBMs. These treatments increased OS and progression-free survival (PFS), indicating potential for future treatments [46].

## 5. Conclusions

The available evidence suggests that GBM origin, growth, and advancement do not solely rely on intrinsic mutations of neoplastic cells. Indeed, tumor genesis and progression seem to be greatly impacted by the surrounding microenvironment and the interactions between tumor and non-tumor cells.

Current literature has shown that the TME and the communication between different cells and factors have an important influence not only on the progression and recurrence, but also on resistance to therapy. In detail, a high proportion of M2 macrophages’, specific miRNAs’ (including miR-1238, miRNA-31, miRNA-221, miRNA-222, miRNA-416a, and miRNA-21) and lncRNAs’ (lnc-TALC, SBF2-AS1, linc-RA1, and AHIF) profiles in the TME may play a pivotal role in the acquisition of TMZ resistance and radioresistance of GBM cells.

Recent studies have highlighted the potential of TME and tumor-associated macrophages (TAMs) as targets for therapeutic interventions. Despite progress in understanding the origin, polarization, and functional diversity of TAMs, the intricate interplay and dynamics between GBM and TAMs remain poorly understood. To successfully target the immunosuppressive M2-like TAM population for GBM treatment, a comprehensive comprehension of the interplay between TAMs and other immune cells within the TME is critical.

Comprehending the bridging role of TAMs between the innate and adaptive immune systems is crucial for enhancing an anti-tumor immune response. TAMs targeting has recently displayed a potential in preclinical trials. Combining TAMs-targeting therapeutics with other immunotherapies in novel synergistic combinations may provide a survival advantage in GBM patients.

## Figures and Tables

**Table 1:** Role of the TME in the promotion and development of chemoresistance in GBM.

Author, Year	Methods	Cell Lines Studied	Mechanism of Resistance
Pessina et al., 2015 [26]	Cellular culture and immunohistochemistry	Murine NK1.1 + CD3-; human GBM GL261	Multidrug-resistance transporter Abcc3 provides TMZ resistance to NK cells
Azambuja et al., 2017 [27]	Cellular culture and immunohistochemistry	Human GBM GL261; human GBM cells–astrocytes LN229	M2 macrophage polarization with high IL-10 release and antioxidant potential of the TME contribute to GBM TMZ resistance
Hide et al., 2018 [14]	Cellular culture and immunohistochemistry	Human GBM A172 and T98G	M2 macrophages produce HB-EGF and IL-1β, conferring TMZ resistance potential on GBM cells
Zhang et al., 2019 [23]	Cellular culture and immunohistochemistry	Human GBM U87, LN229, A172, T98, U251; human embryonic kidney 293 T cells	Exosomal transfer of lnc RNASBF2-AS1 from TME to GBM enhances chemoresistance to TMZ
Yin et al., 2019 [15]	Cellular culture and RT-qPCR	Human GBM U251 and GBM-1	MiR-1238 exosomes levels are higher in TMZ-resistant GBM
Wu et al., 2019 [28]	Cellular culture and RT-qPCR	Human GBM LN229, U251, 551 W, and HG7	TMZ-associated lncRNA in GBM recurrence (lnc-TALC) may play a role in regulating the c-Met signaling pathway, obtained by activation of the Stat3/p300 complex due to competitive binding with miR-20b-3p. This can promote DNA repair enzyme expression of O-methylguanine-DNA methyl-transferase (MGMT) and favor TMZ resistance due to histone H3 acetylation modulation.
Witusik-Perkowska et al., 2019 [29]	Cellular culture and RT-qPCR	Human GBM cell cultures derived from three patients with GBM	TME of in vitro GBM cell cultures changes the profile of specific miRNAs related to tumor drug resistance (miRNA-221, miRNA-31, miRNA-21, miRNA-222)
Chuang et al., 2019 [30]	Cellular culture and RT-qPCR	Human GBM U87MG and LN18	miRNA-21-enriched exosomes from M2 GBM-associated macrophages provide TMZ resistance to GBM; STAT3 Inhibitor Pacritinib can overcome this mechanism of TMZ resistance
Pustchi et al., 2020 [31]	Cellular culture, immunohistochemistry	LN229 GBM cells–astrocytes	GFAP-vimentin and Notch1-survivin signaling in astrocytes of TME are implicated in TMZ resistance of GBM
Li et al., 2021 [32]	Cellular culture genomics	Human microglial HMC3 and murine microglial BV-2	GBM-associated microglia secreted IL11 to activate STAT3-MYC signaling, inducing enhanced TMZ resistance
Xue et al., 2021 [33]	Cellular culture	Human GBM U87 and GBM-1	CD90^low^ GBM-associated mesenchymal stem and stromal cells favor TMZ resistance by the activation of FOXS1-mediated epithelial-mesenchymal transition in GBM cells
Li et al., 2021 [34]	Cellular culture, genomics, and immunohistochemistry	Human microglial HMC3, and murine microglial cell line BV-2	GBM cell-derived lncRNA-containing exosomes induce microglia to produce Complement C5 and develop TMZ resistance
Zhou et al., 2022 [35]	Cellular culture and immunohistochemistry	Human GBM U251, LN229, and U87; human monocyte cell line THP-1 cells	PTEN loss mutation in the macrophages of the TME may be associated with the development of chemoresistance
Zhang et al., 2022 [5]	Cellular culture, genomics, and immunohistochemistry	Human GBM LN229 and U251; THP-1 monocyte-derived macrophages	Hypoxic M2 macrophages can activate the PI3K/Akt/Nrf2 pathway by the secretion of VEGF in GBM cells to favor cancer stemness, aggressiveness, and TMZ resistance.
Liu et al., 2022 [20]	Cellular culture, genomics, and immunohistochemistry	Human GBM U87MG and U251MG; THP-1 monocyte	ADAM8 causes tumor infiltration of tumor-associated macrophages through HB-EGF/EGFR-mediated CCL2 expression and promotes TMZ resistance in GBM

**Table 2:** Role of the TME in the promotion and development of radioresistance in GBM.

Author, Year	Methods	Cell Lines Studied	Mechanism of Resistance
Jamal et al., 2010 [21]	Cellular culture and immunohistochemistry	Human GBM NSC11 and GBMJ1	Orthotopic xenografts GBM cells can show increased capability of repairing DNA double-strand breaks and tend to be less susceptible to induction when compared with cells cultured in vitro, thus promoting the TME as a possible source of GBM radioresistance
Hsieh et al., 2012 [22]	Cellular culture and immunohistochemistry	GBM cell lines GBM8401 and U251	The subunit 4 in NADPH oxidase of the TME can favor GBM cycling hypoxia-promoted radiation resistance
Hide et al., 2018 [14]	Cellular culture and immunohistochemistry	Human GBM A172 and T98G, human GBM cells	Oligodendrocyte progenitor cells secrete FGF1 and EGF, and macrophages produce HB-EGF and IL-1β, conferring stemness radioresistant potential on GBM cells
Dai et al., 2018 [13]	Cellular culture and RT-qPCR	Human GBM U87-MG, U251-MG, A172 and T98G.	LncRNA AHIF promotes GBM progression and radioresistance via exosomes
Zheng et al., 2020 [36]	Cellular culture and RT-qPCR	Human GBM M059J, U251, M059K, and U87	LncRNA linc-RA1 inhibits autophagy and promotes Radioresistance by preventing H2Bub1/USP44 combination in GBM cells
Jang et al., 2022 [37]	Cellular culture and immunohistochemistry	M1/M2 macrophages	M1/M2 macrophage ratios and radiosensitivity are inversely associated: radioresistant TME contain more M2 than M1 macrophages
Zhou et al., 2022 [35]	Cellular culture and immunohistochemistry	GBM cell lines (U251, LN229, and U87) and the human monocyte cell line THP-1 cells	PTEN loss mutation in the macrophages of the TME may be associated with the development of radioresistance

**Table 3:** Literature review on GBM immune TME and immunotherapeutic strategies.

Author, Year	Type of Article	Cell and Pathway Involved	Target Molecules	Drug Tested	Type of Test (Human/Mice/In Vitro)	Results
Chandran et al., 2017 [38]	Review	Tumor associated lymphocytes	CTLA4-PD1	CTLA4-PD1 inhibitors (e.g., Ipilimumab)	In vitro	OS improvement in association with CTX and RT
Roesch et al., 2018 [39]	Review	GAMs	VEGF-VEGFR; IL-10, STAT3	Sunitinib, Bevacizumab; STAT3 inhibitor	In vitro	Increased OS and tumor growth inhibition
Sahin et al., 2018 [40]	Research	Chimeric T Cells	EGFRvIII	Anti-EGFRvIII Chimeric T Cells	In vitro and mice	Increased OS in mice
Goff et al., 2019 [41]	Research	Chimeric T Cells	EGFRvIII	Anti-EGFRvIII Chimeric T Cells	Human (phase I pilot trial)	No OS increment
Li et al., 2019 [18]	Research	GAMs	CDK6	Palbociclib	In vitro	Increased TMZ sensitivity
Flores-Toro et al., 2020 [42]	Research	GSCs	CCR2 and PD-1	CCR2 antagonist CCX872 and anti-PD-1	Mice	Increased OS
Hu et al., 2021 [43]	Research	Cytokines	CXCL1/2	CXCL1/2 inhibitor + TMZ	Mice	Increased OS
Li et al., 2021 [34]	Review	GSCs	SOX2	Zika virus	In vitro	Apoptosis of GSCs
Serpe et al., 2021 [44]	Research	Extracellular vesicles	miRNA-124	miRNA-124 upregulation	In vitro and mice	Tumor mass reduction in vitro and increased OS in mice
Andersen et al., 2021 [45]	Review	GAMs	TNF-α and NF-κB	inhibition of NF-κB signaling	In vitro and mice	Increased infiltration of cytotoxic T cells and decreased tumor growth.
Xun et al., 2021 [46]	Review	TLRs	TL3, TLR7, and TLR8	Hiltonol (TLR3 agonist) and Imiquimod (TLR7 and TLR8 agonist)	Mice	Increase in PFS and OS

Abbreviations: CTX, chemotherapy; GAMs, glioma associated macrophages; GCSs, glioma stem cells; OS, overall survival; RT, radiotherapy; TLRs, toll like receptors; TMZ, temozolomide.

**Table 4:** Review of clinical trials on immunotherapy targeting GAM in GBM (from ClinicalTrials.gov).

Category of Immunotherapeutic Agent	Drug Tested	Combined Therapies	Clinical Trials Registry Identifier	Trial Phase	Patients (*n*)	Reported Biological Response
CSF1R Inhibitors	Pexidartinib	/	NCT01349036	II	38	Monocyte ↓
ANG2 Inhibitors	MEDI3617	Bevacizumab	NCT01248949	I	13	ORR: 0%
	Trebananib	Bevacizumab	NCT01609790	II	130	NR
CXCR4 Inhibitors	Plerixafor	TMZ + RT	NCT01977677	I/II	29	CXCL12 ↑
	Plerixafor	Bevacizumab	NCT01339039	I	26	Lymphocytes ↑, Monocytes ↑ CXCL12 ↑, ANG2 ↓, sMET ↓, IL-8 ↓
CD40 Agonists	APX005M	/	NCT03389802	I	45	NR
	2141-V11	D2C7-IT	NCT04547777	I	30	NR
TLR Agonists	Poly-ICLC (TLR3 agonist)	/	NCT01188096	II	47	50% LGG respond, 25% HGG respond
		RT	NCT00052715	II	31	NR
		TMZ + RT	NCT00262730	II	97	NR
		GAA/TT-Peptide Vaccine	NCT00795457	I	13	91% respond
		Peptide Vaccines	NCT00874861	I	10	55% respond
		IMA950 Peptide Vaccine	NCT01920191	I/II	19	NR
		Dendritic Cell Vaccine	NCT00068510	I	28	TNF-a ↑, IL-6 ↑, Lymphocytes ↑
	HSPPC-96 (TLR4 agonist)	/	NCT02122822	I	20	NR
		/	NCT00293423	II	96	NR
	CpG-ODN (TLR9 agonist)	/	NCT00190424	II	34	No benefit
PD-1 Inhibitors	Nivolumab	/	NCT02017717	III	529	ORR: 7.8%
		/	NCT02550249	II	29	CXCL10 ↑, CCL4 ↑, CCL3L1 ↑
		Ipilimumab	NCT03233152	I	27	No benefit
		Lirilumab	NCT02813135	II	397	NR
	Cemiplimab	Veledimex	NCT04006119		40	NR
	Pembrolizumab	Bevacizumab + RT	NCT02313272		32	NR
		Bevacizumab	NCT02337491		80	ORR:20%

Abbreviations: ORR, objective response rate; NR, not reported; RT, radiotherapy; TMZ, temozolomide. ↑ = increase in production; ↓ = reduction of production.

## Data Availability

The authors confirm that the data supporting the findings of this study are available within the article.
